# The complete mitochondrial genome of *Anas crecca* (Anseriformes: Anatidae)

**DOI:** 10.1080/23802359.2017.1339216

**Published:** 2017-06-13

**Authors:** Qin Zhang, Ying Wang, Renrui Chen, Birong Liu, Xianzhao Kan

**Affiliations:** a The Institute of Bioinformatics, College of Life Sciences, Anhui Normal University, Wuhu, China;; b The Provincial Key Laboratory of Conservation and Exploitation Research of Biological Resources in Anhui, Wuhu, China

**Keywords:** *Anas crecca*, mitochondrial, genome, phylogenetic analysis

## Abstract

The complete mitochondrial genome of *Anas crecca* is 16,596 bp in length. It was predicted to contain 13 PCGs, 22 *tRNA* genes, and 2 *rRNA* genes, and a putative control region. All of the PCGs initiate with ATG, except for *MT-CO1*, *MT-CO2*, and *MT-ND5*, which began with GTG. Four types of termination codons were identified. Phylogenetic analysis shows that *A. crecca* clustered with *Anas acuta*, and the monophyly of the genera *Anser*, *Cygnus*, *Aythya*, and *Anas* was strongly supported. Moreover, our results also support a sister-group relationship between Anas and Aythya.

Anatidae is the largest family in Anseriformes among the three families (Anhimidae, Anseranatidae, and Anatidae). About 173 species in 49 genera are found in this family (Gill and Donsker 2017). *Anas* is a relatively large genus of the family Anatidae (Clements et al. 2016). In this study, an adult *Anas crecca* was collected from Wuhu (31.33°N, 118.38°E) of southeastern China, and the specimen was stored in the herbarium of Anhui Normal University. The total genomic DNA of the *A. crecca* (code Kan-A0147) was extracted from the muscle tissue with standard phenol/chloroform methods. Fourteen PCR and sequencing primers specific to *A. crecca* were designed, and two long overlapping fragments were amplified to avoid the possibility of obtaining nuclear copies of mitochondrial genes (Zhang et al. 2012).

We obtained the complete mitochondrial genome of *A. crecca* (GenBank Accession No. KC771255) in this study. We described 16,596 bp of *A. crecca* mitochondrial genome DNA, including 13 protein-coding genes (PCGs), 22 transfer RNA (tRNA) genes, 2 ribosomal RNA (rRNA) genes, and 1 putative control region. All *tRNA* genes can fold into a typical cloverleaf second structure. All of the PCGs initiate with ATG, except for *MT-CO1*, *MT-CO2*, and *MT-ND5*, which began with GTG. Four types of termination codons were identified, canonical TAA and TAG termination codons are found in eight PCGs, while AGG for *MT-CO1* and *MT-ND1*, the remaining genes (*MT-ND2*, *MT-CO3*, and *MT-ND4*) terminate with an incomplete stop codon (T––). We can detect a single control region (D-loop) between *tRNA^Glu^* and *tRNA^Phe^*. The D-loop region is 1044 bp in length. The overall base composition of the mitochondrial genome is as follows: A (28.99%), T (22.37%), G (15.96%), and C (32.67%), with the A + T content of 51.37%. Nucleotide composition was calculated by MEGA6 (V6.06) (Tamura et al. 2013). The *MT-ND6* gene of *A. crecca* mitogenome has strong skews of T versus A (−0.57) (Kan et al. 2010), and a strong skew of C versus G (0.53). The other 12 PCGs of the *A. crecca* mitochondrial genome have a slight skew of A versus T (0–0.32), and a strong skew of C versus G (GC skew= −0.65 to −0.31).

The phylogenetic position of *A. crecca* was estimated from a concatenated dataset based on 13 PCGs (Kan et al. 2010). Maximum likelihood (ML) analyses were performed with raxmlGUI (V1.3.1) (Silvestro and Michalak 2012; Jiang et al. 2015). Neighbour-Joining (NJ) analyses were conducted in MEGA6 (V6.06), and Maximum parsimony (MP) analyses were calculated using PAUP* (V4.0 b 10) (Swofford 2003). The complete mitochondrial DNA sequences have been used successfully to estimate phylogenetic relationships among Anseriformes. Our results show that *A. crecca* clustered with *A. acuta,* the bootstrap value was 85% in ML analyses. Our results also indicate that the monophyly of *Anser*, *Cygnus*, *Aythya*, and *Anas* was strongly supported, respectively. Moreover, our results found a sister-group relationship between *Anas* and *Aythya*, and *Anseranas* was basal to the balance of the Anseriformes. This study will contribute to the phylogenetic analyses in Anseriformes (Figure 1).

**Figure 1.**
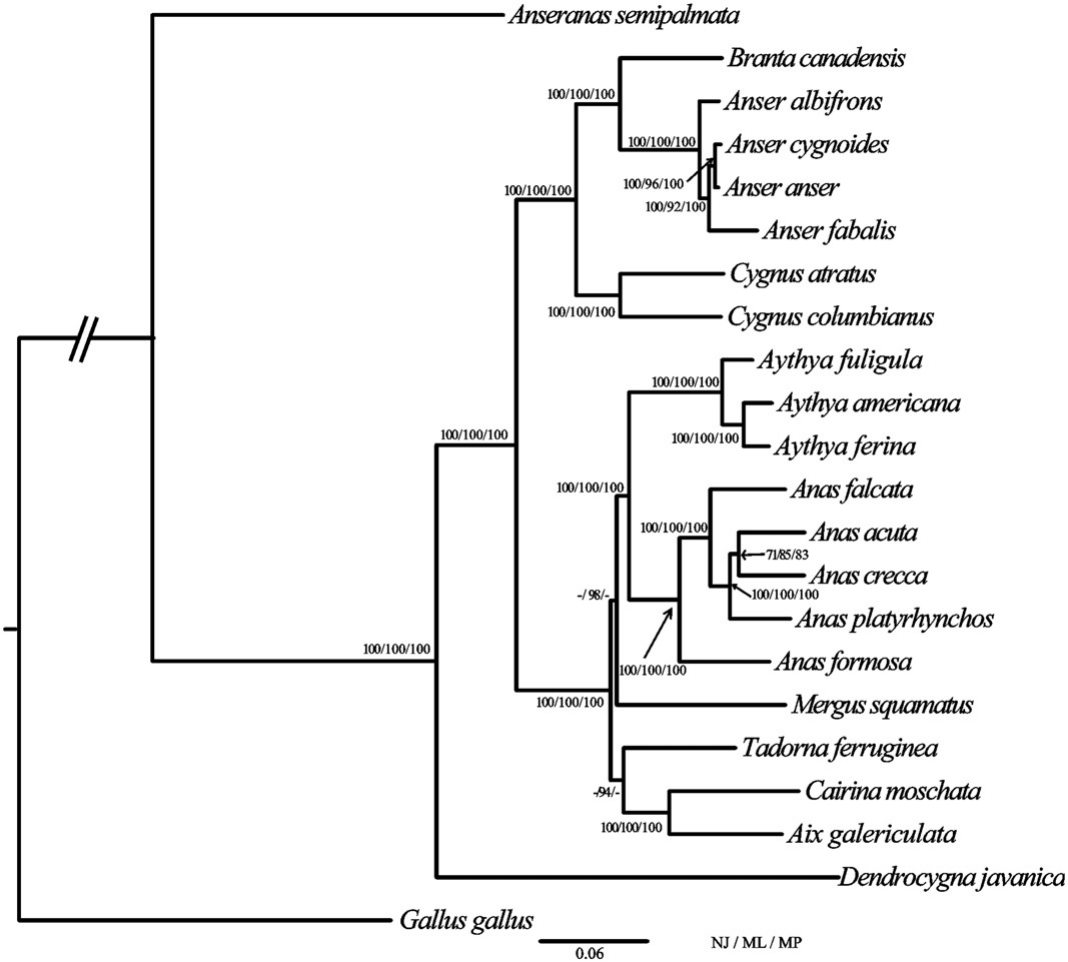
Phylogenetic tree of the relationships among Anseriformes based on the nucleotide dataset of the 13 PCGs. *Gallus gallus* served as outgroup. The NJ analyses were conducted using 1000 bootstrap values in MEGA v 6.06, ML analyses were implemented in ML + rapid bootstrap for 100 replicates under GTRGAMMA, and MP analyses were performed with 1000 bootstrap values. Number of above each node indicates the NJ, ML, and MP bootstrap support values, respectively. Branch lengths and topologies came from the ML analysis. All 22 species' accession numbers are listed as below: *Gallus gallus* KM096864, *Anseranas semipalmata* NC_005933, *Dendrocygna javanica* NC_012844, *Aix galericulata* NC_023969, *Cairina moschata* NC_010965, *Tadorna ferruginea* NC_024640, *Mergus squamatus* NC_016723, *Anas formosa* NC_015482, *Anas platyrhynchos* NC_009684, *Anas acuta* NC_024631, *Anas falcata* NC_023352, *Aythya ferina* NC_024602, *Aythya americana* NC_000877, *Aythya fuligula* NC_024595, *Cygnus columbianus* NC_007691, *Cygnus atratus* NC_012843, *Anser fabalis* NC_016922, *Anser anser* NC_011196, *Anser cygnoides* NC_023832, *Anser albifrons* NC_004539, *Branta canadensis* NC_007011, and *Anas crecca* KC771255.

## References

[CIT0001] Clements J, Schulenberg T, Iliff M, Roberson D, Fredericks T, Sullivan B, Wood C. 2016 The eBird/Clements checklist of birds of the world: v2016. Available from: http://www.birds.cornell.edu/clementschecklist/download/.

[CIT0002] Gill F, Donsker D. 2017. IOC World Bird List (v 7.2). doi: 10.14344/IOC.ML.7.2.

[CIT0003] Jiang L, Chen J, Wang P, Ren Q, Yuan J, Qian C, Hua X, Guo Z, Zhang L, Yang J. 2015 The mitochondrial genomes of *Aquila fasciata* and *Buteo lagopus* (Aves, Accipitriformes): sequence, structure and phylogenetic analyses. PLoS One. 10:e0136297. 26295156 10.1371/journal.pone.0136297 PMC4546579

[CIT0004] Kan X, Li X, Lei Z, Wang M, Chen L, Gao H, Yang Z. 2010 Complete mitochondrial genome of Cabot’s tragopan, *Tragopan caboti* (Galliformes: Phasianidae). Genet Mol Res. 9:1204–1216. 20589618 10.4238/vol9-2gmr820

[CIT0005] Kan X, Yang J, Li X, Chen L, Lei Z, Wang M, Qian C, Gao H, Yang Z. 2010 Phylogeny of major lineages of galliform birds (Aves: Galliformes) based on complete mitochondrial genomes. Genet Mol Res. 9:1625–1633. 20730714 10.4238/vol9-3gmr898

[CIT0006] Silvestro D, Michalak I. 2012 raxmlGUI: a graphical front-end for RAxML. Organ Divers Evol. 12:335–337.

[CIT0007] Swofford DL. 2003. PAUP*. Phylogenetic analysis using parsimony (*and other methods). Version 4.

[CIT0008] Tamura K, Stecher G, Peterson D, Filipski A, Kumar S. 2013 MEGA6: molecular evolutionary genetics analysis version 6.0. Mol Biol Evol. 30:2725–2729. 24132122 10.1093/molbev/mst197 PMC3840312

[CIT0009] Zhang L, Wang L, Gowda V, Wang M, Li X, Kan X. 2012 The mitochondrial genome of the Cinnamon Bittern, *Ixobrychus cinnamomeus* (Pelecaniformes: Ardeidae): sequence, structure and phylogenetic analysis. Mol Biol Rep. 39:8315–8326. 22699875 10.1007/s11033-012-1681-1

